# College and Hospital Notes

**Published:** 1899-07

**Authors:** 


### COLLEGE AND HOSPITAL NOTES.

---

AT THE Buffalo General Hospital Dr. Regina Flood Keyes has been formally appointed assistant gynecologist on the attending staff. A new position has been created, termed assistant to surgical clinic, and Dr. Edward J. Meyer has received the appointment.

---

THE announcement was made two weeks ago of changes in the staff at St. Mary's Maternity and Infant Asylum, on Edward Street, whereby the institution came under the medical and surgical management of the staff of the Buffalo Sisters of Charity Hospital. Dr. L. G. Hanley is announced as chief obstetrician; the attending obstetricians are Drs. Nelson W. Wilson, Pierce J. Candee, C. J. Carr and D. V. McClure. Dr. W. H. Slacer is at the head of the medical staff. His assistants are Drs. Joseph P. Burke and James Stoddart.

---

THE Woman's Medical College of the New York Infirmary for women and children, celebrated its last annual commencement May 25, 1899. It had been previously announced that the college was to go out of existence and the reasons for the step were announced by the board of trustees as follows :

The constant tendency of the medical as well as other professional schools has been toward a university connection, until at the present time each of the other important medical schools in New York is the medical department of a university. The medical college has been in existence for forty years and until last year has offered to women the only means of medical education in New York. It has now fulfilled its purpose and medical education may hereafter be obtained by women in New York in the same classes, under the same faculty and with the same clinical opportunities as men.

Under these circumstances, the trustees have considered it unadvisable to continue longer the maintenance of an independent school, and believe that the medical education of women can be better served and more speedily advanced by recognising the inevitable result of competition with the university schools.

---

A MARINE hospital station has been proposed at Buffalo. The surgeon-general of the marine hospital service at Washington favors the project and it is hoped and believed that the Hon. D. S. Alexander, M. C., of Buffalo, will introduce a bill on the subject at the next session of congress.

